# The impact of COVID-19 on the number of active small primary care businesses by severity of the pandemic: evidence from South Korea

**DOI:** 10.1186/s12875-022-01676-0

**Published:** 2022-04-04

**Authors:** Kyung-Bok Son

**Affiliations:** College of Pharmacy, Hanyang University, 55 Hanyangdeahak-ro, Sangnok-gu, Ansan, Gyeonggi-do 15588 South Korea

**Keywords:** COVID-19, Primary care, Primary care business, South Korea

## Abstract

**Background:**

Health systems have become financially fragile owing to the economic recession caused by the COVID-19 pandemic. However, small primary care businesses have received less policy attention than public health and secondary care. We aimed to estimate the impact of COVID-19 on the number of active small primary care businesses in South Korea.

**Methods:**

We selected clinics, dental clinics, oriental clinics, and pharmacies as primary care businesses. Our estimation took advantage of regional variations in COVID-19 cases in South Korea. We determined the number of active primary care businesses from 2019 1Q to 2021 1Q on a quarterly basis, and conducted interrupted time series analysis to estimate the effects of COVID-19 on this sector.

**Results:**

This study found no significant increase or decrease in the number of clinics, dental clinics, and oriental clinics immediately after the pandemic began or in the time trends after the pandemic. However, there was a significant increase in the number of pharmacies immediately after the pandemic. The most affected area presented different trends in the number of pharmacies, dental clinics, and oriental clinics.

**Conclusions:**

Impact of the pandemic on the number of active small primary care business were low in South Korea. However, the impact varied according to the type of primary care setting and severity of the pandemic. The additional public health role of primary care could be associated with the sustenance of primary care businesses.

**Supplementary Information:**

The online version contains supplementary material available at 10.1186/s12875-022-01676-0.

## Background

The Director-General of the World Health Organization (WHO) on January 30, 2020 declared that the coronavirus disease 2019 (COVID-19) constituted a Public Health Emergency of International Concern [1]. By July 27, 2021, the global number of confirmed cases of COVID-19 had reached 194 million and the number of deaths had increased to 4 million, while a total of 3.696 billion vaccine doses had been administered [2]. In South Korea, the number of confirmed COVID-19 cases and deaths had reached 191,531 and 2079, respectively, by July 27, 2021 [2]. As the number indicates, COVID-19 has caused a substantial burden on health systems worldwide [3–5].

COVID-19 has had a tremendous impact on the economy [6–8] as well as public health [3–5]. Governments have devised various interventions to prevent the spread of COVID-19 in order to protect their health systems from being overwhelmed [9–16]. The interventions range from social and physical distancing restrictions [9–11] to government-mandated stay-at-home rules [12, 13], business closures [13, 14], and lockdowns [15, 16]. These measures, combined with the fear of infection, have inevitably led to radical shifts in contemporary lifestyles and working habits [17, 18]. These interventions have intentionally or unintentionally contributed to an economic recession [19, 20]. It is reasonable to expect the pandemic to have a negative impact on business. Globally, difficulties in accessing goods and services, decreased sales and revenue, business closures, and widespread layoffs due to the pandemic have been reported [21].

Health systems have not been immune to the economic recession. It is a well-documented fact that health care utilization, including physician consultations, specialist referrals, and hospital admissions, has decreased during the pandemic [22–24]. Note that the decrease in health care utilization is due to the overwhelm generated by the pandemic, not to lack of demand. Furthermore, the pandemic has exacerbated the precariousness of small businesses around the world [25, 26]. The number of small businesses in the United States decreased substantially during the COVID-19 outbreak [27]. In a survey of primary care physicians in the United States, 6% reported the closure of their practice and 35% reported layoffs [28]. In South Korea, the majority of primary care businesses, including clinics, dental clinics, oriental clinics, and pharmacies, are privately owned and small-scale businesses in terms of financial resources [29], implying that the nation’s primary care business is financially fragile. Furthermore, primary care has received less policy attention than public health and secondary care [30], and studies investigating the effects of COVID-19 on small primary care businesses are scarce. This study sought to estimate the impact of COVID-19 on the number of active small primary care businesses in South Korea. To this end, we determined the number of active primary care businesses from 2019 1Q to 2021 1Q on a quarterly basis and conducted interrupted time series analysis to estimate the effects of COVID-19 on this sector.

### The COVID-19 pandemic in South Korea and three areas

Supplement 1 describes basic characteristics of three areas, including metropolitan, the most affected, and the remaining area. The first case of COVID-19, a passenger who arrived at Incheon International Airport, Seoul from Wuhan, China, was detected on January 20, 2020 [31]. Within a month after the first case was detected, the cumulative number of cases increased slightly. There have been four peaks so far, with the nation currently witnessing the fourth peak. The first, second, third, and fourth peaks were observed in February 2020, August 2020, November 2020, and July 2021, respectively.

The first peak was associated with Shincheonji Church, with over 5000 cases linked to the church after the first church member tested positive on February 18, 2020 [31]. The event caused cluster infections among members and non-members of the church in Gyeongsangbuk-do Province and Daegu. Until March 31, 2020, the accumulated number of COVID-19 cases in these areas was 7984, or nearly 82% of the total of 9786 cases. Therefore, we defined these areas as the most affected area during the initial stage of the pandemic (the most affected area). The second peak was related to the demonstration of far-right groups and the Sarang Jeil church in Seoul [32]. The protesters violated the quarantine regulations and gathered in Gwanhwamun Plaza in Seoul on August 15, 2020. After the demonstration, the number of COVID-19 cases in Seoul, Incheon, and Gyeonggi-do Province increased substantially. The number of cases in these areas as a percentage of the overall cases increased from 22% (2860/12,800) on June 30, 2020, to 46% (9496/20,584) on September 30, 2020. We defined these areas as the metropolitan area. The third and fourth peaks were consequences of the relaxation of the social and physical distancing regulations [32]. On March 31, 2021, the number of accumulated cases in the metropolitan area, the remaining area, and the most affected area were 62,808 (66%), 20,607 (22%), and 12,085 (13%), respectively.

## Methods

### Study overview

This study determined the number of active primary care businesses from 2019 1Q to 2021 1Q on a quarterly basis and conducted interrupted time series analysis to estimate the effects of COVID-19 on this sector. We selected (physician) clinics, dental clinics, oriental clinics, and pharmacies as primary care businesses. These clinics and pharmacies are privately owned and provide primary care services under the National Health Insurance [29]. Our estimation took advantage of regional variations in COVID-19 cases in South Korea. Regional variations in COVID-19 cases and the case fatality rate were policy concerns in South Korea [31]. During the initial stage of the outbreak, a rapid surge in COVID-19 cases occurred in the most affected area. On March 26, 2020, the total number of patients in the most affected area as a percentage of all COVID-19 cases in the country was 82% (7984 out of 9786). The following two characteristics provided grounds for a natural experimental study design. The most affected area became a “ghost city” without lockdowns in the area. The concentration of COVID-19 cases in the most affected area was not associated with any other underlying social and economic factors.

### Data sources

The Health Insurance Review and Assessment Service (HIRA) provides information on all health care institutions in South Korea on a quarterly basis for the last 3 years. We requested HIRA to provide related data from 2019 1Q to 2021 1Q. The data listed all kinds of health care institutions with detailed information on their names and locations.

### Statistical analysis

We aggregated the number of primary health care institutions on a quarterly basis, separated the institutions according to their location, and then re-aggregated the number of institutions by location. As we already explained, locations were classified as the metropolitan area, the most affected area, and the remaining area. To describe time trends in the number of institutions, we calculated the compounded annual growth rate (CAGR) during the study period.

$$CAGR=\left\{{\left(\frac{EV}{BV}\right)}^{\frac{1}{n}}-1\right\}\ \mathrm{X}\ 100$$

EV: Ending Value. BV: Beginning Value. N: Number of periods.

Then, we applied interrupted time series analysis to estimate the effects of COVID-19 on primary care businesses. The coefficients of β_0_, β_1_, β_2_, and β_3_ indicated the baseline level of the institutions, the trends in the number of institutions before the pandemic, the immediate effect of the pandemic, and the trends in the number of institutions after the pandemic began, respectively [33]. The error term ε_t_ indicates deviation from the fitted model [34]. We conducted Durbin-Watson test to detect the presence of autocorrelation at lag 1 in the error term and accepted null hypothesis (true autocorrelation is 0). Thus, we applied ordinary least squares regression, which provides no adjustment for autocorrelation [35]. Data management and analysis were performed using R statistical software (version 4.1.2). Statistical significance was noted when *p*-values were less than 0.05.

$${\mathrm{Y}}_{\mathrm{t}}={\upbeta}_0+{\upbeta}_1{\mathrm{T}}_{\mathrm{t}}+{\upbeta}_2{\mathrm{D}}_{\mathrm{t}}+{\upbeta}_3{\mathrm{P}}_{\mathrm{t}}+{\upvarepsilon}_{\mathrm{t}}$$

Y_t_: the aggregated number of health care institutions.T_t_: a continuous variable indicating the quarter that has passed from the start of the study period.D_t_: a dummy variable indicating the pre-pandemic period (0) or intra-pandemic period (1) (2020 1Q).P_t_: a continuous variable indicating the time that has passed since the occurrence of the pandemic.

## Results

### Number of primary care business owners

**Fig. 1:**
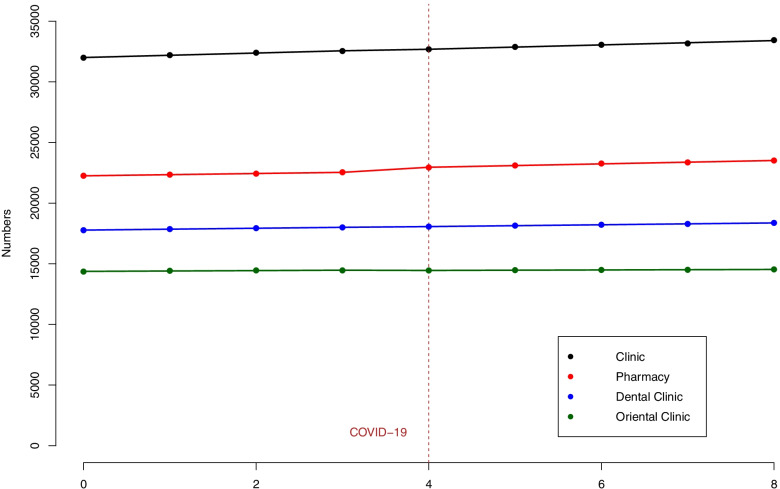
Number of primary care institutions from 2019 1Q to 2021 1Q on a quarterly basis

Figure 1 describes the changes in the number of primary care institutions from 2019 1Q to 2021 1Q. There were 31,992 clinics, 22,258 pharmacies, 17,768 dental clinics, and 14,354 oriental clinics in 2019 1Q. The numbers increased to 33,442 clinics, 23,514 pharmacies, 18,372 dental clinics, and 14,536 oriental clinics in 2021 1Q. During the study period, pharmacies had the highest CAGR (2.78%), followed by clinics (2.24%), dental clinics (1.69%), and oriental clinics (0.63%).

**Fig. 2:**
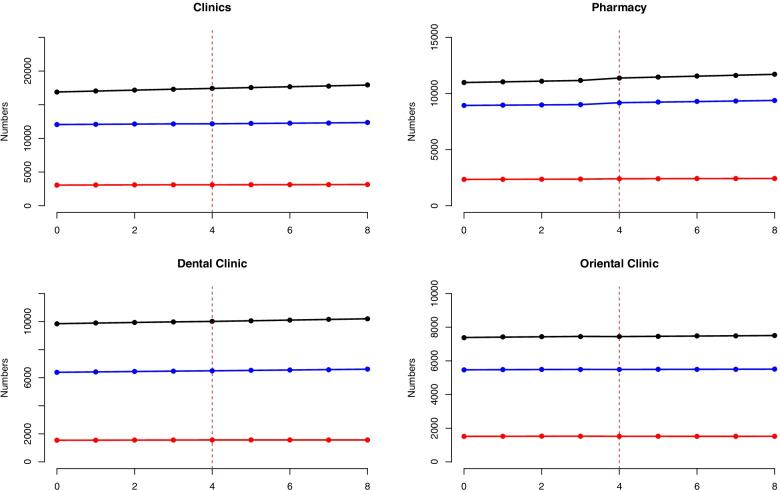
Number of primary care institutions categorized into three areas from 2019 1Q to 2021 1Q on a quarterly basis. Note: Black, blue, and red curve indicates the number of institutions in the metropolitan area, the remaining area, and the most affected area, respectively

Fig. 2 describes the changes in the number of primary care institutions categorized into three areas: the metropolitan area, the most affected area, and the remaining area. In 2019 1Q, about 53% of the 31,992 clinics were distributed across the metropolitan area, 38% across the remaining area, and 10% in the most affected area. In 2021 1Q, the respective distribution percentages of the 33,442 clinics were 54, 37, and 9%. The CAGR during the study period was highest for the metropolitan area (3.09%), followed by the most affected area (1.41%) and the remaining area (1.11%). Similarly, 49% of the 22,258 pharmacies were distributed across the metropolitan area, 40% across the remaining area, and 11% across the most affected area in 2019 1Q; the distribution percentages across the three areas did not change in 2021 1Q. The CAGR during the study period was highest in the metropolitan area (3.28%), followed by the remaining area (2.44%) and the most affected area (1.73%).

### Effects of COVID on small primary care business owners

Table 1 presents the main results of the interrupted times series analysis. A significant increase in the time trends before the pandemic was observed for clinics (185.1, *p* <  0.0001), pharmacies (95.0, *p* <  0.0001), dental clinics (76.6, *p* <  0.0001), and oriental clinics (32.8, *p* <  0.0001). The immediate effect of the pandemic and the time trends after the start of the pandemic were not significant for the number of clinics, dental clinics, and oriental clinics. However, the immediate effect of the pandemic (279.9, *p* = 0.0001) and the time trends after the start of the pandemic (44.8, *p* = 0.0084) were significant for pharmacies.

**Table 1:** Main results of interrupted time series analysis

	Coefficient	SE	*P*-value
Clinic			
Time	185.1	15.99	< 0.0001
Event	− 53.3	47.98	0.3170
Time Since Event	−5.8	19.59	0.7790
Constant	32,008.1	29.92	< 0.0001
Pharmacy			
Time	95.0	8.70	0.0001
Event	279.9	26.12	0.0001
Time Since Event	44.8	10.66	0.0084
Constant	22,253.5	16.29	< 0.0001
Dental clinic			
Time	76.6	3.81	< 0.0001
Event	−14.9	11.43	0.2490
Time Since Event	−1.2	4.66	0.8070
Constant	17,776.1	7.13	< 0.0001
Oriental clinic			
Time	32.8	7.33	0.0065
Event	−41.2	22.01	0.1201
Time Since Event	−12.4	8.98	0.2261
Constant	14,371.8	13.72	< 0.0001

Table 2 presents the results of the interrupted time series analysis by area. In the case of clinics, we found results that were consistent with the main analyses. The number of clinics increased significantly in the three areas before the pandemic. The immediate effect of the pandemic and the time trends after the start of the pandemic were not significant. In the case of pharmacies, we found results that were not consistent with the main analyses. When we separated pharmacies by location, we found that the number of pharmacies increased significantly immediately after the pandemic in the metropolitan area (135.6, *p* = 0.0001), the remaining area (120.5, *p* = 0.0002), and the most affected area (23.8, *p* = 0.0045). Furthermore, the time trends after the start of the pandemic were significant in the metropolitan area (21.6, *p* = 0.0084) and the remaining area (26.1, *p* = 0.0038). We found interesting results for the most affected area. The time trends for the number of dental clinics after the start of the pandemic presented a significant decrease, and the immediate effect of the pandemic on the number of oriental clinics also presented a significant decrease.

**Table 2:** Results of interrupted time series analysis by area

	Metropolitan area			The most affected area			The remaining area		
	Coefficient	SE	*P*-value	Coefficient	SE	*P*-value	Coefficient	SE	*P*-value
Clinic									
Time	137.0	8.66	< 0.0001	15.7	2.06	0.0006	32.4	6.17	0.0033
Event	−7.3	25.99	0.790	−13.3	6.20	0.0850	−32.7	18.51	0.1375
Time Since Event	−12.1	10.61	0.306	−5.8	2.53	0.0707	12.1	7.55	0.1702
Constant	16,891.0	16.21	< 0.0001	3060.7	3.87	< 0.0001	12,056.4	11.54	< 0.0001
Pharmacy									
Time	61.0	4.19	< 0.0001	9.6	1.62	0.0019	24.4	4.20	0.0021
Event	135.6	12.58	0.0001	23.8	4.87	0.0045	120.5	12.61	0.0002
Time Since Event	21.6	5.13	0.0084	−2.9	1.99	0.2051	26.1	5.14	0.0038
Constant	10,977.0	7.84	< 0.0001	2341.1	3.04	< 0.0001	8935.4	3.04	< 0.0001
Dental clinic									
Time	43.1	2.69	< 0.0001	5.2	1.53	0.0193	28.3	2.76	0.0001
Event	−14.9	8.08	0.125	5.2	4.58	0.3086	−5.2	8.30	0.5586
Time Since Event	3.8	3.29	0.301	−5.1	1.87	0.0417	0.1	3.39	0.9776
Constant	9851.1	5.03	< 0.0001	1540.7	2.86	< 0.0001	6384.3	5.17	< 0.0001
Oriental clinic									
Time	21.1	4.56	0.0057	3.9	1.82	0.0860	7.8	2.45	0.0244
Event	−24.5	13.7	0.1337	−8.3	5.48	0.0190	−8.4	7.35	0.3049
Time Since Event	−5.6	5.59	0.3626	−3.7	2.23	0.1590	−3.1	3.00	0.3490
Constant	7389.1	8.54	< 0.0001	1513.4	3.419	< 0.0001	5469.3	4.58	< 0.0001

## Discussion

COVID-19 has exacerbated the precariousness of small businesses around the world [25–27]. Health systems have not been immune to the economic recession. However, primary care has received less policy attention than public health and secondary care [30], and studies investigating the effect of COVID-19 on small primary care businesses are scarce. This study estimated the impact of COVID-19 on small primary care businesses in South Korea.

### Interpretation of the study findings

In the current study, we noticed that the number of primary care businesses had continuously increased before the pandemic. There was no significant increase or decrease in the number of clinics, dental clinics, and oriental clinics after the start of pandemic or in the time trends after the start of the pandemic. However, the number of pharmacies increased significantly immediately after the start of pandemic. When we conducted additional analyses by areas, we found a heterogeneous impact of COVID-19 on small primary care businesses in the South Korean settings. The most affected area presented different trends in the number of pharmacies, dental clinics, and oriental clinics.

We used the aggregated number of institutions for analysis. Four scenarios were possible for the individual institutions. First, business owners continuously ran their business during the study period. Second, the original owners closed their business, which was taken over by other persons. Third, owners closed their business without someone’s taking over. Fourth, owners started a new business without taking over someone else’s business. The first and second scenarios do not influence the total number of institutions, while the third and fourth do. If the number in the fourth category exceeds that in the third category, the total number of institutions would increase. Thus, the increase in the number of pharmacies indicates that the number of new pharmacies exceeded that of closed pharmacies without someone’s taking over.

### Non-declining trends in the number of primary care businesses

In the United States, physicians running small businesses reported steep declines in the number of patients and practices [23]. Even though the government has lifted some of the restrictions imposed on non-COVID-19 care, the decline in revenue is not expected to bounce back to pre-pandemic levels in the foreseeable future [28]. Furthermore, in a survey of 558 primary care physicians in the United States, 6% reported a closure of their practice and 35% reported layoffs [28]. In contrast to the scenario in the United States, we found no decrease in the number of primary care businesses in South Korea.

This interesting trend in South Korea can be explained by several factors. First, the South Korean government has successfully responded to the pandemic. From the initial stage of the outbreak, the government has successfully adopted a test, trace, and treat strategy with extensive utilization of digital technology to cope with the pandemic [32]. The number of COVID-19 cases and case fatality rates in South Korea have been within the capacity of the health system [31]. The case fatality rate in South Korea (1.79%; 183/10,237) was lower than that in the United States (2.45%; 6856/279,512) on March 31, 2020. The gaps between the two countries widened on June 30, 2020 (2.16%; 283/13,089 versus 4.68%;131,124/2,804,392) [2]. The successful response to the pandemic might be associated with a relatively less affected economy or fast economic recovery.

Second, the pandemic most likely did not significantly affect health care utilization, thus helping sustain primary care businesses. In South Korea, total outpatient visits and pharmacy visits for picking up prescribed medication in 2020 1Q decreased by 8.28 and 6.86%, respectively, compared to 2019 1Q. However, the total cost of outpatient visits decreased only by 0.31% while that of pharmacy visits increased by 3.62% during the same period [36]. These figures indicate that patients visited health care institutions less frequently during the pandemic, but the average cost per visit increased significantly. Similarly, in Canada, the number of small businesses in the health sector increased by 11.4% in the first 5 months of 2020 compared to 2019 [13]. Other sectors such as art, culture, recreation, and sports (− 14.8%), education, law, and government services (− 13.6%), and sales and service (− 12.8%) witnessed a decrease in the number of small businesses [13].

Third, small primary care businesses have been adjusting in various ways to the changing business environment caused by the pandemic. Starting new clinics or pharmacies requires substantial investment during the initial stage [37, 38]. The average start-up cost for a small business was 102 million Korean Won (KRW) in 2019 [37], while the estimated average start-up cost for a clinic ranged from 300 million KRW to 500 million KRW [38]. Given the substantial sunk cost, closing their business might be difficult for owners of clinics and pharmacies. They would reduce the number of employees instead of closing their business.

### Increasing trends in the number of pharmacy businesses

The number of pharmacies increased immediately after the start of the pandemic, and the time trends after the start of the pandemic still presented positive values. These typical trends could be explained by the characteristics of pharmacies in South Korea.

First, starting a new pharmacy business has been characterized by seasonality. The national examination for acquiring a pharmacist license is conducted in January every year, and the results are released in February [39]. In 2009, the curriculum for the College of Pharmacy was reorganized from a four-year system to a six-year system [39]. Students who had completed the second year of college or higher were eligible to take the pharmacy education eligibility test (PEET) [39]. The new curriculum required at least two additional years to become a pharmacist. As it takes more time and resources to be a pharmacist, the majority of newly licensed pharmacists made conservative choices in their careers after graduation [40]. Furthermore, the current role of pharmacists is limited to medicine-centered services [41], implying that the majority of newly licensed pharmacists initiate their career at community pharmacies and some start their own pharmacy business. Thus, the increased number of pharmacies immediately after the start of the pandemic can be partially explained by the graduation effect. Approximately, 2000 pharmacists were newly licensed in February 2020 [39].

Second, the South Korean government assigned new roles to pharmacies during the initial stage of the pandemic. Securing, distributing, and wearing face masks are critical prevention strategies for coping with the pandemic. The government supported the production of face masks in response to the surge in demand, and released a “plan on managed distribution of face masks” on March 5, 2020, introducing the concept of “public masks” [42]. From March 6 to May 31, 2020, pharmacies were in charge of the “fair” distribution of face masks. In particular, public masks were distributed at pharmacies, and a purchase limit of two public masks per person per week was introduced. The purchase limit was managed by a drug utilization review system installed at pharmacies. The newly added public health role of pharmacies seemed to offset the decline in pharmacy sales during the pandemic.

### Study limitations

This study has several limitations. First, we used aggregated data to estimate the impact of COVID-19 on the number of active small primary care businesses in South Korea. Owing to the lack of micro-level data, the characteristics of the institutions and their owners were not considered in this study. Second, closing decisions and/or coping strategies against the pandemic might be associated with the financial and human resources of the institutions [13]. However, these variables were not controlled in this study. Third, we used the number of active primary care businesses as the dependent variable. The variable could not entirely capture the impact of the pandemic on primary care businesses. The pandemic might have driven changes in the revenue and employment model of primary care businesses [23]. Fourth, South Korea has successfully controlled the spread of COVID-19 [31]. Therefore, findings from this study cannot be generalized to other countries where the pandemic has had detrimental effects on their health systems. Furthermore, countries with a national health system might present different effects of the pandemic on primary care business [30]. Finally, the business environment changes rapidly, primarily based on the situation of the pandemic. As we already mentioned, there were four peaks in February 2020, August 2020, November 2020, and July 2021, respectively, with the fourth peak currently ongoing. The economic effect of the pandemic on small primary care businesses depends on the interaction between the intensity and the duration of the pandemic.

## Conclusions

Health systems are financially fragile owing to the economic recession caused by the pandemic. However, small primary care businesses have received less attention than public health and secondary care. We estimated the impact of COVID-19 on the number of small primary care businesses in South Korea using an interrupted time series analysis. Our estimation took advantage of regional variations in the severity of the pandemic, which provided grounds for a natural experimental study design. Impact of the pandemic on the number of active small primary care business were low in South Korea. However, the impact varied according to the type of primary care setting and severity of the pandemic. The additional public health role of primary care could be associated with the sustenance of the number of primary care businesses.

## Supplementary Information

**Additional file 1: Supplement 1.** Basic characteristics of three areas, including metropolitan, the most affected, and the remaining area.

## Data Availability

The data-sets used and/or analysed during the current study available from the corresponding author on reasonable request.
