# Supplementary material for: The impact of COVID-19 on the number of active small primary care businesses by severity of the pandemic: evidence from South Korea
Source: BMC Prim Care. 2022 Apr 4;23:67. doi: 10.1186/s12875-022-01676-0 (PMC8978167; doi:10.1186/s12875-022-01676-0)
Supplement: Supplementary file 1 — Additional file 1: Supplement 1. Basic characteristics of three areas, including metropolitan, the most affected, and the remaining area. [file 12875_2022_1676_MOESM1_ESM.docx]

Supplement 1. Basic characteristics of three areas

|  | Metropolitan area | | The most affected area | | The remaining area | |
| --- | --- | --- | --- | --- | --- | --- |
| Region | Seoul, Incheon, and Gyeonggi-do | | Daegu and Gyeongsangbuk-do | | The remaining area | |
| Land area (km^2^) | 11,865 | 12% | 19,917 | 20% | 68,631 | 68% |
| Population (1,000 persons) | 25,958 | 50% | 5,074 | 10% | 20,749 | 40% |
| Population Density  (persons/km^2^) | 2,188 |  | 255 |  | 302 |  |
|  |  |  |  |  |  |  |
| Health care institutions  (Excluding pharmacies) | 34,882 | 52% | 6,514 | 10% | 25,704 | 38% |
| Hospital beds | 252,296 | 36% | 80,340 | 12% | 364,322 | 52% |
|  |  |  |  |  |  |  |
| COVID-19 cases |  |  |  |  |  |  |
| 2020 1Q | 990 | 10% | 7,984 | 82% | 812 | 8% |
| 2020 2Q | 2,860 | 22% | 8,295 | 65% | 1,645 | 13% |
| 2020 3Q | 9,496 | 46% | 8,544 | 42% | 2,544 | 12% |
| Cases per 1000 persons |  |  |  |  |  |  |
| 2020 1Q | 0.038 |  | 1.574 |  | 0.039 |  |
| 2020 2Q | 0.110 |  | 1.635 |  | 0.079 |  |
| 2020 3Q | 0.366 |  | 1.684 |  | 0.123 |  |
| 2020 4Q | 1.300 |  | 1.963 |  | 0.516 |  |
